# Lymphocyte surface expression patterns differ in inflammatory and infectious stages in polytraumatized and septic shock patients

**DOI:** 10.1186/cc13419

**Published:** 2014-03-17

**Authors:** M Weiss, L Brach, M Georgieff, F Gebhard, M Huber-Lang, M Schneider

**Affiliations:** 1 University Hospital Medical School, Ulm, Germany

## Introduction

Lymphocytes show different cell surface molecules depending on the degree of their activation or suppression. We used this surface expression to look at inflammatory or infectious states in patients with polytrauma and patients with sepsis. The present study was performed to clarify: what is the expression profile of lymphocyte surface markers in polytraumatized patients over time; and are there differences in the expression patterns of polytraumatized patients compared with healthy controls and with patients with severe sepsis and septic shock?

## Methods

In a prospective observational study in a surgical ICU, surface expression of CD3, CD4, CD8, IL7R, CD4/25, CD8/25, CD2/86, CD28, CD3/56, CD2, CD39, CD244, CD11b, and CD56 on lymphocytes was measured by flow cytometry. We collected data from six healthy controls, from eight patients with severe sepsis or septic shock, and, longitudinally from eight polytraumatized patients on admission (0 hours), and at 4, 12, 24, 48, 120, and 240 hours. ISS, SAPS 3 and SOFA score reflect severity of injury, of disease and of organ dysfunctions of the polytraumatized patients.

## Results

In septic and polytraumatized patients, the expression of CD3, CD28 and CD2 was significantly lower than in controls. In polytraumatized patients, surface expression of CD244, CD39 and CD8/25 was significantly higher than in controls. Within the polytraumatized patients, we found two different subgroups, group A with septic progression and Group B without septic progression of disease. Especially, the septic polytraumatized patients revealed very low levels of CD3, CD2 and cD28. Moreover, their levels of CD39 and CD8/25 were less increased and their ISS higher than those of the nonseptic subgroup.

## Conclusion

Surface expression molecules of polytraumatized patients differ from those of the healthy controls and within the group. The more heavily injured polytraumatized patients are more likely to develop severe sepsis and septic shock with surface expression profiles on lymphocytes comparable with those of septic patients.

